# Broadening perspectives on trauma and recovery: a socio-interpersonal view of PTSD†

**DOI:** 10.3402/ejpt.v7.29303

**Published:** 2016-03-18

**Authors:** Andreas Maercker, Tobias Hecker

**Affiliations:** Division of Psychopathology and Clinical Intervention, Department of Psychology, University of Zurich, Zurich, Switzerland

**Keywords:** Post-traumatic stress disorder, interpersonal processes, social context, disclosure, social sharing

## Abstract

Posttraumatic stress disorder (PTSD) is one of the very few mental disorders that requires by definition an environmental context—a traumatic event or events—as a precondition for diagnosis. Both trauma sequelae and recovery always occur in the context of social–interpersonal contexts, for example, in interaction with a partner, family, the community, and the society. The present paper elaborates and extends the social–interpersonal framework model of PTSD. This was developed to complement other intrapersonally focused models of PTSD, which emphasize alterations in an individual's memory, cognitions, or neurobiology. Four primary reasons for broadening the perspective from the individual to the interpersonal–societal contexts are discussed. The three layers of the model (social affects, close relationships, and culture and society) are outlined. We further discuss additional insights and benefits of the social–interpersonal perspective for the growing field of research regarding resilience after traumatic experiences. The paper closes with an outlook on therapy approaches and interventions considering this broader social–interpersonal perspective on PTSD.

Within the traditional professional perspective of clinical psychology and psychiatry, posttraumatic stress disorder (PTSD) is usually considered solely in terms of the individual. Approaches that aim to describe, explain, or treat PTSD or other stress- and trauma-related disorders center on the patient and do not consider the social context in which they are situated (e.g., families, partners, close friends, communities, or even societies). This focus on the individual perspective has its value and benefits. This is particularly evident when we consider the progress in evidence-based treatments for PTSD over the last 20 years. However, this individual-centered perspective also leaves out a host of potential influential factors external to the individual. The influence of family members, peers, and the society at large all have an impact on the development and maintenance of the individual's PTSD symptoms. Thus, focusing only on the individual when treating PTSD may not be enough to ensure an optimal chance of recovery. In reaction to this, some prominent researchers have sought to question or broaden the individual-centered approach to explain and treat PTSD (Ajduković & Ajduković, 2003; Somasundaram, 2014; Summerfield, 1999). In a previous theoretical and review paper, our group (Maercker & Horn, 2013) developed a framework model on social–interpersonal processes in PTSD to complement the other existing models of memory or neurobiological dysfunctions in PTSD. The present paper will further elaborate the theory of our social–interpersonal model, with the addition of new findings, and present some applications for both clinical and psychosocial work with survivors of trauma.

## Broadening the focus on trauma survivors

There are several reasons to expand the study of trauma—both theoretical and clinical: 1) the philosophical view that humans are social beings; 2) the well-articulated view that often entire societies rather than individuals are traumatized; 3) the occurrence of traumatic stress on a global scale; and 4) the improved but still limited effectiveness of individualized psychotherapies for PTSD.

Many diverse philosophical theories converge upon the same fundamental insight that the social, or interpersonal realm, is a central element of human nature (Aristotle, 1999; Ricoeur, 2005). This is implicit in any consideration of mental disorders, mental health, and psychological well-being. As human beings, we can be individual agents, acting independently as well as group members (e.g., family, neighborhood, society), acting interdependently. Whether we act independently or interdependently varies depending on different factors, for example, historical era, culture, and individual life-span phase. Interdependent psychological functioning, defined as the degree to which members of the group are mutually dependent on the others, has recently gained increased attention in social psychology and psychiatry, indicating a broad range of neurobiological and psychological covariation of this distinction (Han & Ma, 2014). Even in individualistic European or Westernized societies, the degree of sociality or interdependence should not be neglected—and this is particularly true for PTSD. A landmark meta-analysis demonstrated this finding that “(perceived) social support” as the most important variable was negatively related to PTSD symptom severity (Brewin et al., 2000). This relation was replicated for both perceived and received social support in another meta-analysis (Prati & Pietrantoni, 2010).

The term “traumatized societies” has received more attention in recent research. It is defined as a large social group sharing the same geographical territory and dominant cultural values that have experienced genocide, war, or other extensive violence, so that the majority of the society's members experienced exposure to extremely threatening or horrific events. An incomplete list of such societies includes the territory or at least larger parts of Afghanistan, Bosnia-Herzegovina, Chechnya, Democratic Republic of Congo, Eastern Ukraine, Eritrea, etc. This term has also been used for populations that have either survived historical trauma, or are descended from those who have, for example, Jewish Holocaust survivors, Native Americans, and Apartheid witnesses. Several authors, however, questioned the usefulness of the term “traumatized societies”, for example, because it is often used too unspecifically, without giving detailed descriptions of its characteristics (Bruner, 2014). Large-scale trauma may also lead to disruption of the very fabric of a society (e.g., dismantling of institutions, displacement of potential support networks). Therefore, a cautious consideration of broad- or long-lasting effects of trauma should include the societal context as well.

Data from international aid organizations [related to United Nations (UN) or non-governmental organizations] indicate that traumatic events are ubiquitous. Every minute approximately 10 children die due to hunger and malnutrition, which sums up to 6 million deceased children per year (UNICEF, 2012); the daily death toll due to violent conflict around the world sums up to around 500 persons (www.crisisgroup.org); and in the first 6 months of 2015, around 2,500 individuals died by drowning in the Mediterranean Sea while trying to reach Europe from the North African coast (Amnesty International, 2015). All these deceased have close family and friends who most probably mourn their deaths and may have simultaneously experienced what the trauma criterion in DSM-5 and ICD-10 define as traumatization by witnessing. Altogether, this would entail there being millions of traumatized individuals, particularly in war-torn countries and regions. A growing amount of research has highlighted a substantial gap, particularly in resource-poor countries, between the burden caused by mental disorders and the resources devoted to prevent and treat them (Collins, Insel, Chockalingam, Daar, & Maddox, 2013). The consequence is that more than 75% of people with trauma-related and other mental health disorders do not receive any official mental health care at all in these countries. It is unlikely that individual trauma-focused therapy could be mobilized to deal with this great number of trauma survivors for a number of reasons, for example, lack of trained therapists, lack of resources, and lack of a mental health care infrastructure. Therefore, the World Health Organization (WHO) increasingly advocates for larger scale programs in post-conflict regions, targeting whole communities or whole societies (Epping-Jordan et al., 2015; Tol et al., 2014).

In the developed countries, with their contemporary standards of mental health sciences and service opportunities, PTSD and related disorders can be treated in the majority of patients suffering from these conditions. An enthusiastic and tremendously inventive community of scientists and practitioners has developed effective treatment approaches for PTSD, as indicated, for example, by prominent publications by the International and the European Society for Traumatic Stress Studies (Foa, Keane, Friedman, & Cohen, 2008; Olff et al., 2015; Schnyder et al., 2015). However, we also know that up to 40% of our traumatized patients nevertheless do not reap all the benefits from our individualized PTSD therapies (Bradley, Greene, Russ, Dutra, & Westen, 2014). Current treatment guidelines only adhere to treatments targeted to modify individual dysfunctions in memory, cognition, or affect. However, there are few new approaches aimed at facilitating social support or restructuring communities or societies (see closing section of this article for more detail).

## The social–interpersonal framework model of PTSD

**Fig. 1:**
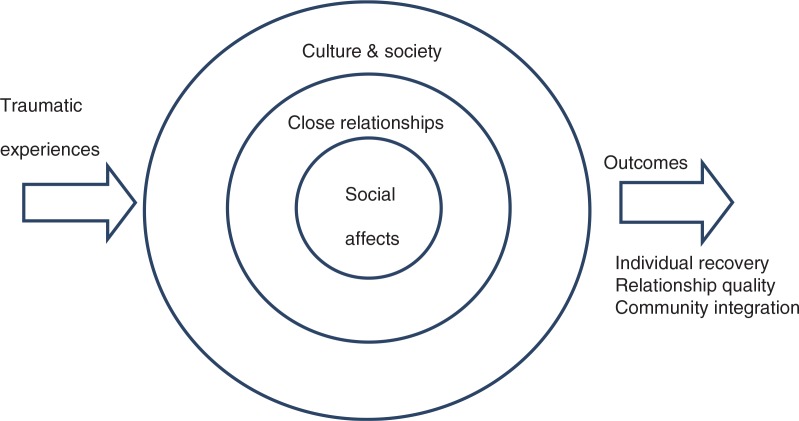
The social–interpersonal framework model for trauma sequelae. (From Maercker & Horn, 2013.)

It is vital to understand how all the various interpersonal and social factors relevant for the development and maintenance of PTSD fit together. To achieve this, Maercker and Horn (2013) developed a model as an explanatory framework. It consists of factors that determine how traumatic stress is mitigated or intensified by different layers of person–environment interactions. The simplified structure is shown in Fig. 1 (a more detailed version of this figure is given in Maercker & Horn, 2013). The model describes three layers: 1) social affects comprising shame, guilt, anger, revenge, etc.; 2) close relationships including trauma disclosure, social support or negative exchange, empathy, etc.; and 3) culture and society, comprising aspects like the collective experience of trauma, social acknowledgment as victim or survivor, cultural value orientation, etc.

### Social affects

The first level of social affects comprises well-described modes of action tendencies and interactive styles of traumatized persons. Affective reactions that relate to other persons, groups, or communities are termed “social affects” (Hareli & Parkinson, 2008). For example, guilt is generally considered to function for its own immediate relief. However, chronic guilty feelings contribute to the maintenance of psychopathology in the long run (Rachman, 1993). The functionality of shame in the context of PTSD is still unclear. Shame has been demonstrated to be strongly related to intrapersonal avoidance (Street, Gibson, & Holohan, 2005). Furthermore, it is plausible that shame is related to social withdrawal (for more details, see Maercker & Horn, 2013). Whereas posttraumatic shame and guilt have been investigated frequently, other social affects, such as anger or aggression, have received little attention despite their common manifestation in trauma survivors. In continuation of Maercker and Horn's (2013) descriptions, we focus here on recent findings on anger and aggression in trauma survivors. The association between PTSD and interpersonal aggression is a robust finding in the literature. When a person experiences repeated and constant threats to their life, this person may develop cognitive networks that guide cognitions, emotions, and actions in order to increase chances of survival (Elbert & Schauer, 2002). Although being highly alert and aroused in dangerous situations has survival advantages, in situations where threat to life is low, it can produce inappropriate and aggressive behavior. High PTSD symptom severity was, for example, associated with reactive aggression—an aggressive reaction to some perceived provocation or threat—in survivors of hurricane Katrina (Marsee, 2008) or in veterans (Byrne & Riggs, 1996). A meta-analysis of 31 studies on the association between PTSD and intimate relationship discord found medium-size associations between PTSD and interpersonal physical and psychological aggression, with trait anger mediating this relationship (Taft, Watkins, Stafford, Street, & Monson, 2011).

Furthermore, it has been repeatedly shown that a person's own experiences of childhood maltreatment and their own victimization through intimate partner violence predict aggressive parenting behavior (Saile, Ertl, Neuner, & Catani, 2014). These findings provide further evidence that exposure to trauma and violence in the past is associated with the display of aggressive behavior and violence. Correspondingly, former child soldiers who have been frequently exposed to severe violence also perpetrated more types of violence (Weierstall, Schalinski, Crombach, Hecker, & Elbert, 2012). These findings, however, were not limited simply to reactively aggressive acts that grew out of trauma-related suffering. Instead, they reported that exposure to violence fostered appetitive aggression. For this subtype of aggression, the infliction of harm upon an individual is itself rewarding, fascinating, and a source of enjoyment —above and beyond secondary rewards like status or material benefits (Hecker, Hermenau, Maedl, Elbert, & Schauer, 2012). In environments dominated by violence, exposure to violence and other traumatic stressors have been linked to appetitive aggression and encouraged violent behavior, thereby creating a cycle of violence (Hecker, Fetz, Ainamani, & Elbert, 2015). Developing appetitive aggression seems to be an adaptive survival strategy for children growing up in such violent environments (Hermenau, Hecker, Maedl, Schauer, & Elbert, 2013). Appetitive aggression has been shown to be a major risk factor for future violent behavior. Therefore, both reactive and appetitive aggression may hinder successful treatment and integration into civil society (Annan, Brier, & Aryemo, 2009). Thus, social affects such as anger, aggression, and revenge need to be addressed to enable trauma survivors to fully reintegrate into their society.

### Close relationships

The second layer comprises trauma-relevant processes in close relationships of the survivors. Disclosure of traumatic experiences is a phenomenon that deserves particular attention. If a trauma survivor is able to share parts or even central elements of his or her horrible experience with family and friends, he or she reports better psychological well-being. This basic relationship has been described by clinicians (Briere & Scott, 2013; Gray et al., 2012) as well as in so-called analog research designs, that is, with non-traumatized students who were studied after experimental manipulation as if they were traumatized (Pennebaker, 1995). Disclosure prevents “oppressive silence”, which may extend from close relationships into broader societal contexts—as already described by several pioneering traumatic stress studies (e.g., Boston Women's Health Book Collective, 2011; Shay, 1994). Recent research on disclosure in PTSD incorporated methodological advancements of longitudinal as well as dyadic assessments. Pielmaier, Milek, Nussbeck, Walder, and Maercker (2013) studied patients and their significant others after severe traumatic brain injury at 3, 6, and 12 months post-injury. They measured three aspects of dysfunctional disclosure tendencies (reluctance to talk, urge to talk, emotional overreactions) based on previous research (Mueller, Mörgeli, & Maercker, 2008). As hypothesized, the extent of PTSD symptom severity in patients was related both with their own dysfunctional disclosure, that of their significant other, and additionally with the interaction between the two disclosure styles. If both were frank to each other (low dysfunctional disclosure), they reported low PTSD symptom severity and vice versa with high dysfunctional disclosure and high PTSD symptom severity. If the significant others showed highly dysfunctional disclosure while the patient showed low levels of dysfunctional disclosure, medium levels of PTSD were reported, indicating the crucial role of the significant others in fostering healing through verbal exchange.

Regarding social support or negative social exchange, important lines of research originate from PTSD research on the consequences of natural disaster. Kaniasty and Norris (2004) describe how initial positively received social support shortly after the traumatic event—in a phase they call “honeymoon stage”—often transfers into a negative state of social support deterioration for various reasons, for example, interpersonal weariness (“pressure cooker effect”), need for actual support surpasses the availability, potential for interpersonal conflicts, lack of consensus in appraisal of the event, etc. In a 2-year longitudinal study on severe flood victims, they showed by means of elaborated cross-lagged analysis that social support is the most prominent cause of a decrease in symptoms during the earlier phase after a trauma (Kaniasty & Norris, 2008). However, the causal relationship appears to switch directions 18 months after the trauma, when an increase in trauma-related symptoms leads to less reported social support. This finding strongly indicates the need for sequentially ordered social interactions and avoids regarding social support as a self-revolving inexhaustible positive resource.

### Culture and society

The third level of the social–interpersonal model represents the societal and cultural sphere. Thus far, traumatic stress responses have predominantly been studied in individualistic contexts. Yet, whether collectivistic or interdependent orientations may lead to different symptom-society dynamics remains unclear. In addition to the clinical and research specifications in the Maercker and Horn (2013) article, we focus subsequently on new results on the issues of social acknowledgment as a victim or survivor as well as on human value orientation changes. Social or societal acknowledgment as a victim is assumed to be particularly relevant for survivors of man-made trauma; it is close to the restorative justice perception of persons trying to overcome the traumatic disruption of their lives (Sullivan & Tifft, 2007). Maercker and Müller (2004) have developed a 16-item questionnaire measuring the three independent subscales of recognition, family disapproval, and general disapproval. Studies with this questionnaire were conducted, for example, with war victims in Poland, Germany, Indonesia, and the USA (e.g., Lis-Turlejska, Szumial, & Okuniewska, 2012; Schumm, Koucky, & Bartel, 2014), refugees (Maercker, Povilonyte, Lianova, & Pöhlmann, 2009); former political prisoners from East Germany or Lithuania (e.g., Kazlauskas & Želvienė, 2015); and traumatized street gang members in South Africa (Sommer et al., submitted). Across the various cross-sectional studies, the correlation between the total score of self-perceived social acknowledgment and PTSD ranged between *r=*−0.25 and −0.45. In longitudinal studies, the score predicted a regression Beta coefficient of up to −0.33, indicating a large effect of social acknowledgment—or its lack, that is, disapproval—on trauma sequelae (e.g., Mueller et al., 2008).

Human value orientations are rarely considered in clinical psychology or psychiatry as a relevant phenomenon, probably because they are regarded as impersonal and abstract dimensions. This disregard is unfortunate, because value orientations span a variety of important social and psychological phenomena, from intrapersonal concerns to regulatory matters of the whole society and culture (Boer & Fischer, 2013). Intrapersonally, values guide individual motivation by setting goals or proscriptions for managing one's life. Regarding societies or cultures, value orientations regulate, for example, morality, ideas of progress or endeavor, and social order (Schwartz, 1994). Very few studies have been conducted on value orientations in trauma- and stress-disorder victims. Those that have been carried out indicate that modern (e.g., self-direction, stimulation, hedonism) and traditional values (e.g., power, achievement, conformity) differentially mediate the relationship between trauma exposure, social support process, and the extent of PTSD symptom severity, with—non-trivially—stronger traditional values worsening, and higher modern values ameliorating the health outcomes (Maercker, Forstmeier, Wagner, Glaesmer, & Brähler, 2008; Mueller, Orth, Wang, & Maercker, 2009). Zimmermann et al. (2014) confirmed this pattern for German soldiers after recent NATO deployment in Afghanistan. This is in contrast to conventionally held beliefs that personal orientations towards traditional values have in general a positive impact on health (cf. Kleinman & Kleinman, 1985).

A systematic survey of European studies on mental health and PTSD (Wittchen et al., 2011) together with the data from the European Social Survey (www.europeansocialsurvey.org, round 1, 2002) allowed an exciting exploratory data analysis on probable value change after war exposure. In this study, Burri and Maercker (2014) analyzed national PTSD prevalence rates in European countries and examined the complex interrelations between this and countries’ pattern of value orientations, rates of war death (in WWII and the Yugoslavian wars), and the interaction between values and war death rates. The results indicate a strong interaction between these variables and the temporal sequence of association between war deaths and subsequent value orientations (measured around the year 2000). The findings suggest a value change from modern to traditional values in countries with higher war death tolls (Burri & Maercker, 2014). If this result can be confirmed by other data, it would explain the decrease of societal support for individual rights observed by various scholars for post-conflict regions (Ajduković & Ajduković, 2003; Somasundaram, 2014). This would be one of the most far-reaching consequences of traumatization.

## Resilience as application of the social–interpersonal model

Yet, many survivors of traumatic experiences do not develop PTSD or other trauma-related disorders. Furthermore, the spontaneous remission rate in this group is high. Therefore, many clinicians and researchers in the field of traumatic stress studies are fascinated by recent developments concerning human resilience. Resilience as the capacity to bounce back from a negative experience can be formulated on both an individual (biological, psychological, etc.) and social–interpersonal level. In recent research, the former has been more popular, whereas the latter is often disregarded. However, at least one prominent paper on resilience, written for a broader audience, the American Psychological Association's “10 ways to build resilience” lists a social–interpersonal feature as no. 1 in its outline: “Make connections. Good relationships with close family members, friends or others are important. Accepting help and support from those who care about you and will listen to you strengthens resilience. Some people find that being active in civic groups, faith-based organizations, or other local groups provides social support and can help with reclaiming hope. Assisting others in their time of need also can benefit the helper” (American Psychological Association, 2012). Yet, face validity is not always supported by empirical evidence. Therefore, further research is highly required to gain a better understanding of resilience factors on a social–interpersonal level.

In resilience research, a leading author in particular promotes what he calls the “socio-ecological model of resilience” (Ungar, 2011). This model highlights crucial aspects of resilience, like facilitative environments, which enable the individual access to resources, the interaction of protective mechanisms with exterior risk factors, and a longitudinal period of record as well as cultural relativity. Ungar's research is primarily concerned with resilience in children confronted with challenging environments. He argues, for example, that for children from conflict areas, exposure to violence during war was less debilitating for their well-being than the separation from their caregivers (e.g., Solomon & Laufer, 2005). Research on community-level resilience factors in adults, however, is still sparse. Maercker, Hilpert, and Burri (2015) applied the socio-ecological resilience model to a group of Swiss elderly people with a long-term history of childhood trauma and adversity. They confirmed the potential of these extended approaches by showing, for example, social–interpersonal factors outperforming the well-established individual resource of high self-esteem in predicting resilience.

## Outlook for therapy and interventions

The present paper should not end without a short overview on the efforts to overcome trauma sequelae on an interpersonal and societal level. In brief, the main goals of such interventions, apart from the reduction of PTSD symptoms, are improvements of relationship and community functioning. In the logic of the presented social–interpersonal model (see Fig. 1), social affect focused therapies are necessary for its first layer, couple and family therapies, for its second layer, and community interventions for the third layer.

### Social affects

Trauma-related guilt and shame is thoroughly addressed in evidenced-based individual-level PTSD interventions (Schnyder et al., 2015). Anger and aggression, however, have been a particular focus in traumatized soldiers and veterans, although other populations also deserve attention. Yet, research on aggression and anger even within military populations is sparse. For example, in their review of treatment approaches in military populations focusing on anger and aggression, Morland, Love, Mackintosh, Greene, and Rosen, (2012) identified only two formal anger treatment protocols that have been studied in military samples, both of which used multicomponent cognitive behavioral therapy–based protocols to address anger. One study found significant decreases in anger symptoms; whereas the other found significant decreases in both state and trait anger symptoms, as well as reports of physical aggression (see Morland et al., 2012).

Furthermore, Narrative Exposure Therapy for Forensic Offender Rehabilitation (FORNET) aims at reducing both symptoms of traumatic stress and controlling readiness for aggressive behavior (Hecker, Hermenau, Crombach, & Elbert, 2015). FORNET follows the logic of the evidence-based trauma-focused Narrative Exposure Therapy (Schauer, Neuner, & Elbert, 2011) with special emphasis on aggressive behavior and violent acts in the past and future. The first randomized-controlled trials with veterans and violence-affected youths have proven the feasibility of FORNET and found first evidence of a positive outcome, for example, recovered mental health (PTSD, depression), fewer offenses committed, less drug intake, and improved integration into civil society (Crombach & Elbert, 2015; Hermenau, Hecker, Schaal, Maedl, & Elbert, 2013; Köbach, Schaal, Hecker, & Elbert, in press).

### Close relationships

Monson, Wagner, Macdonald, and Brown-Bowers (2015) give an overview on evidence-based couple and family interventions for traumatized family members. In ascending order of focusing trauma or PTSD, they distinguish four classes of interventions: programs on education and family-facilitated engagement, generic couple therapy, partner-assisted intervention, and disorder-specific couple therapy. The class of disorder-specific couple therapy for PTSD has the strongest empirical support in terms of achieving multiple outcomes (i.e., reductions in PTSD, improvements in relational functioning, improvements in partners’ psychological functioning). If both the client and the partner are willing to participate in a dyadic intervention for PTSD, disorder-specific couple therapy is recommended, regardless of level of relationship distress, because these interventions have been tested with couples across the spectrum of relationship satisfaction. The authors acknowledge that these interventions are in their infancy, and additional research is warranted to further establish the effectiveness of the interventions, as the vast majority of the work thus far has been done within the veteran populations (Monson et al., 2015).

### Culture and society

For community-level trauma interventions, it is more difficult to get a comprehensive overview of available services and programs that are developed around the globe. Many of these programs have been developed by local or regional stakeholders that are not as well connected as the communities of clinicians or researchers working on individual trauma sequelae. The WHO more recently tried to integrate and evaluate such posttrauma or post-disasters services to avoid uncoordinated and disproportional aid activities that sometimes spontaneously develop after international emergency situations and to collect systematic knowledge on previous missions (Epping-Jordan et al., 2015; Tol et al., 2014).

Interventions that aim at improving the community situation after traumatic events—and that not only apply large-scale individual treatment—give people a voice (e.g., against local officials, their government); encourage action against deterioration of social support over time; fight embitterment or defeatism; and facilitate identity, value, and meaning reconstruction (Linden & Maercker, 2011; Somasundaram, 2014). The following five classes of programs or services have been developed and applied:

- *Post-disaster mental health programs*: Developed primarily after natural catastrophes, for example, floods, hurricanes, and volcanic eruptions (Norris, Friedman, & Watson, 2002)
- *Community training in critical contexts*: Developed for communities with ongoing high levels of violence, for example, in US Black local communities (Laborde, Magruder, Caye, & Parrish, 2013)
- *Reconciliation and trauma-healing approaches in post-war settings*: For example, applied in post-war Burundi and Rwanda after the genocide (Staub, Pearlman, Gubin, & Hagengimana, 2005; Yeomans, Forman, Herbert, & Yuen, 2010)
- *Post-war community-based rehabilitation programs*: Developed in various regions of the world, for example, in the Balkans (Ajduković & Ajduković, 2003) and in Sri Lanka (Somasundaram & Sivayokan, 2013)
- Mental health reforms advocated by international agencies like the UN and WHO to raise the awareness of psychosocial needs and improve sustainable services for community members in need (Epping-Jordan et al., 2015)

So far little is yet known about the effectiveness of these community-based approaches. Though they are frequently implemented in war and crisis regions, they still lack support from studies using scientifically rigorous methods (Tol et al., 2011). Future research is needed to empirically test their effectiveness.

It is our conviction that the best care for traumatized human beings combines services for the individual, his or her core social group, and the society. The field of traumatic stress studies has been very successful in assisting the individual—but needs to be broadened to encompass family, community, or societal means and measures for recovery or restoration. The individual-centered focus on the individual survivor may not be enough, particularly in regions of war and conflict or after mass disasters.
